# Implementing a nurse-delivered cognitive behavioural therapy intervention to reduce the impact of hot flushes/night sweats in women with breast cancer: a qualitative process evaluation of the MENOS4 trial

**DOI:** 10.1186/s12912-023-01441-3

**Published:** 2023-09-15

**Authors:** Cherish Boxall, Deborah Fenlon, Carl May, Jacqui Nuttall, Myra S. Hunter

**Affiliations:** 1 Southampton Clinical Trials Unit, University of Southampton, Southampton, SO16 6YD UK; 2 Department of Nursing, School of Health and Social Care, Faculty of Medicine, Health and Life Science, University of Swansea, Swansea, UK; 3 Faculty of Public Health and Policy, London School of Hygiene and Tropical Medicine, London, UK; 4 Department of Psychology, Institute of Psychiatry, Psychology and Neuroscience, King’s College London, London, UK

**Keywords:** Nurse, CBT, Implementation, Breast cancer, Group, Qualitative, Evaluation, Theory, Hot flush, Night sweats

## Abstract

**Background:**

Hot flushes and night sweats are life-altering symptoms experienced by many women after breast cancer treatment. A randomised controlled trial (RCT) was conducted to explore the effectiveness of breast care nurse (BCN)-led group cognitive behavioural therapy (CBT). This paper reported findings from a qualitative process evaluation to optimise the CBT intervention and explore the determinants of implementation into routine practice.

**Methods:**

Qualitative process evaluation occurred in parallel with the RCT to explore patient and healthcare staff experiences and perspectives using semi-structured interviews pre-and post-intervention. Normalisation Process Theory (NPT) informed data collection, analysis, and reporting of findings. The analysis involved inductive thematic analysis, NPT coding manual and subsequent mapping onto NPT constructs.

**Results:**

BCNs (n = 10), managers (n = 2), surgeons (n = 3) and trial participants (n = 8) across six recruiting sites took part. All stakeholders believed group CBT met a need for non-medical hot flushes/night sweats treatment, however, had little exposure or understanding of CBT before MENOS4. BCNs believed the work fitted with their identity and felt confident in delivering the sessions. Despite little understanding, patients enrolled onto group CBT because the BCNs were trusted to have the knowledge and understanding to support their needs and despite initial scepticism, reported great benefit from group-based participation. Both managers and surgeons were keen for BCNs to take responsibility for all aspects of CBT delivery, but there were some tensions with existing clinical commitments and organisational priorities.

**Conclusions:**

Both healthcare staff and patient participants believe BCN-led group CBT is a beneficial service but barriers to long-term implementation into routine care suggest there needs to be multi-level organisational support.

**Trial registration:**

NCT02623374 – Last updated 07/12/2015 on ClinicalTrials.gov PRS.

**Supplementary Information:**

The online version contains supplementary material available at 10.1186/s12912-023-01441-3.

**Table:** 

**Text box 1. Contributions to the literature**
• This research will generate evidence for developing future patient-centred CBT.
• This research will inform the development of nurse-led services to be further tested in hybrid effectiveness-implementation trials.
• Findings from this evaluation report implementation determinants that can help the development of future implementation strategies to bridge the gap between research and practice.

## Background

Up to 85% of women experience hot flushes, also known as hot flashes, and night sweats after breast cancer treatment that can continue for more than 5 years and have a significant impact on daily life and quality of sleep [1].

A structured cognitive behavioural therapy (CBT) programme was trialled as an additional service outside of existing usual care as a phase III individually randomised controlled trial (RCT) vs. treatment as usual with a formal process evaluation (called MENOS4) [2]. The CBT was based on the MENOS CBT protocol for menopausal hot flushes/night sweats [3, 4]. The MENOS CBT protocol has been effective in reducing the impact of these symptoms in several RCTs of women going through the menopause transition and for women who have had breast cancer and in different treatment modalities, including group, self-help and online CBT. In the current study, CBT was delivered by trained breast care nurses (BCNs) to patients in a group setting. BCNs were chosen to deliver the intervention because they had the potential to provide a cost-effective solution and were deemed favourable in a previous MENOS trial [5]. Furthermore, group CBT has been shown to result in greater improvements in mood and quality of life, compared to self-help CBT in a study of women going through the menopause transition [6].

BCNs received CBT training from a clinical psychologist over two consecutive days (6 h per day) as close to the first patient group as possible, with an optional telephone top-up. The training consisted of evidence-based information and practical skills to run the group CBT sessions by examining how behaviour and thinking can have a significant impact on women’s experience of hot flushes/night sweats and help develop strategies with the women to manage them. Supportive delivery material was provided in the form of presentation slides, handouts, and notes, including a sleep diary and relaxation CD, which the nurses also completed as part of their training, from a published treatment manual [7].

MENOS4 results demonstrated that CBT is effective in relieving hot flushes/night sweats in women who have had breast cancer [8]. There was a significant (46%) reduction in the mean hot flush/night sweat problem rating score from randomization to 26 weeks in the CBT arm compared with a 15% reduction in the usual care arm. Secondary outcomes, including frequency of hot flush/night sweat frequency, sleep, anxiety, and depression all improved significantly. These results suggest that specialist nurses can be trained to deliver CBT effectively for the alleviation of troublesome menopausal symptoms in women following breast cancer in a clinical setting. However, CBT for these women is not widely available and is rarely offered to women suffering from these symptoms in routine practice [2].

This process evaluation aimed to explore the experiences of key stakeholders involved in MENOS4 and how and in what ways the intervention was implemented to understand influential contextual factors on nurse-delivered CBT. The overarching purpose of the process evaluation was to understand the potential feasibility of routine care implementation. Normalization Process Theory (NPT) [9, 10] was used to interpret the data and facilitate an understanding of how the intervention could be normalised in UK National Health Service (NHS) hospitals.

## Methods

### Evaluation design

**Fig. 1:**
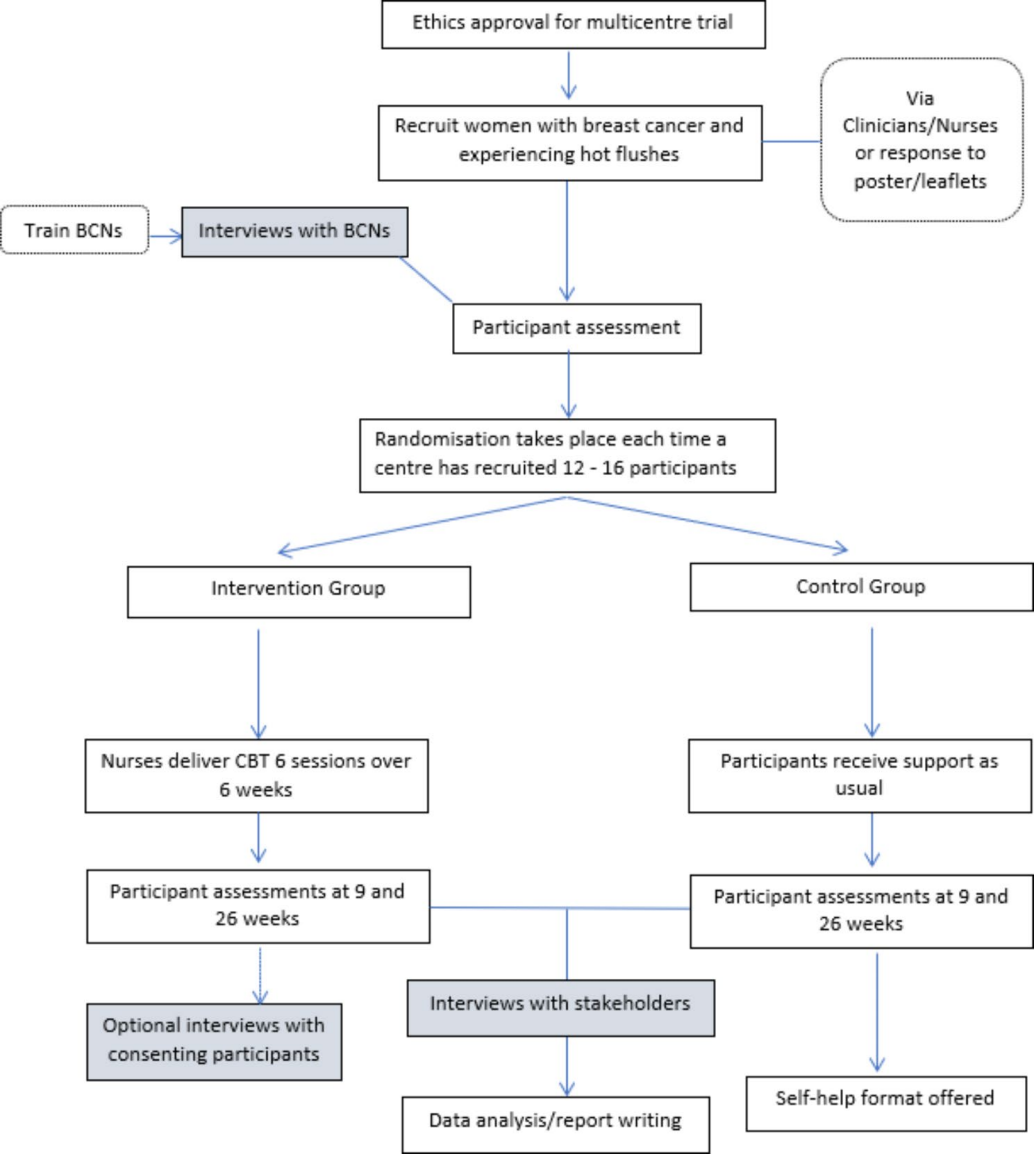
MENOS 4 Trial Schema with qualitative evaluation work highlighted in blue boxes

This was a parallel qualitative process evaluation within a multi-centre phase III RCT (Fig. 1). The RCT individually randomised patient participants to weekly group CBT lasting 90 min for six weeks or usual care to measure the effectiveness of reducing the impact of HFNS in women with breast cancer. Individual participant randomisation meant that all recruiting sites and associated staff interviewed had the experience of implementing nurse-delivered CBT. For the full MENOS4 RCT protocol refer to Fenlon et al. (2018) [11], for more information on the effectiveness of MENOS4 refer to Fenlon et al., 2020 [2].

Post-intervention semi-structured interviews with staff stakeholders including BCNs, managers and surgeons (also referred to as medics) were conducted to explore experiences of delivering the intervention, including barriers and facilitators to implementation and how they were tackled. Post-intervention interviews also took place with trial participants to gain insight into the personal experience of group CBT.

The trial was approved by the University of Southampton and received a favourable opinion from the Health Research Authority Research Ethics Committee and has followed standards for reporting qualitative research (SRQR) guidelines (supplementary material file 1).

### Choice of normalization process theory for process evaluation

The intervention was implemented as part of a trial, where sites agreed to run the trial for an agreed period. Due to this, social processes that influence implementation were prioritised over outer context influencers such as the economic or political context. Normalization process theory (NPT) is a theory of implementation that consists of four constructs; coherence, cognitive participation, collective action, and reflexive monitoring (Table 1). The theory of implementation was used here to highlight and address the challenges and development of strategies for translating research into practice. NPT was chosen over other theories (e.g. consolidated theory for implementation research [12]) because of the implementation context, and its focus on understanding and explaining how the thinking, enacting and organisation of work is operationalised by stakeholders and how these factors facilitate or impede the normalisation of interventions in routine practice [13]. NPT was operationalised in this study through the data collection, sense-making, and reporting of findings.

**Table 1:** NPT constructs with descriptions

NPT construct	Description
Coherence	Individual and collective sense-making people do when faced with implementing a practice
Cognitive Participation	Relational work that people do to build and sustain a community of practice around the intervention
Collective Action	Operational work people to enact practices (e.g. an intervention)
Reflexive Monitoring	Appraisal work that people do to assess and understand what, if any, affect the practice has had on them and the people around them

### Participants

To receive a holistic view of the social process, those involved in the set-up, oversight and/or delivery of the intervention were invited to interview. Purposively sampled groups of interview participants were categorised into the following three groups.

BCNs who delivered the CBT sessions were invited to interview after training and before their first workshop to understand the implementation context (e.g. work dynamics, patient involvement, job responsibilities) and then again from the completion of a six-week CBT group to understand the impact and outcomes of intervention implementation (e.g. impact on existing services).

Managers with organisational responsibility and surgeons responsible for breast care services (both thought to have a potential impact on implementation) were identified through the BCNs and with permission, were invited to interview.

All consenting MENOS4 patients receiving the CBT intervention from each centre were also approached on completion of their sessions to explore their experiences and perspectives of participation, including barriers and facilitators to initial participation and ongoing engagement.

### Data Collection

Post-intervention interviews were conducted between June and July 2018 by an experienced female research fellow working with the core research team, who was not involved in the delivery of the RCT. With consent, they were recorded on a dictaphone and transcribed verbatim where identifiers were replaced with pseudonymised identities. Interviews lasted approximately 30 min and took place at varying times once the intervention was complete. Interviews were conducted using NPT-informed topic guides (supplementary material file 2–4).

#### Breast care nurses

BCN interviews focused on their role, including relation to women with menopausal symptoms, prior understanding of CBT, the experience of delivering group CBT (including recruitment) and support required to incorporate the work to deliver the CBT into their existing workloads.

#### Manager

Topics covered with managers included the processes of encouraging CBT take-up, how patients access it, the potential for running the intervention in routine practice, the extent to which CBT for hot flushes/night sweats is a priority and whether it will continue beyond the trial.

#### Surgeons

Surgeon interviews covered current role, understanding of CBT, relationships with BCNs, post-intervention approaches for helping patients with hot flushes/night sweats, indirect human (time, staffing) and physical (room booking, consumables) resources for running group CBT, and the potential for running the intervention in routine practice.

#### MENOS4 participants

Lastly, patients were interviewed to gain an understanding of their experiences of participating in the CBT group, including motivation for enrolling on the trial.

### Data analysis

Data analysis was completed by CB, a research fellow with two years of qualitative research experience, independent of the interviews and RCT. To enhance coding reliability, the first two transcripts were double-coded by the interviewer, and the data analysis process was supervised by two senior researchers, DF and MH. Transcripts were read for error checking and familiarity purposes and identifying transcript content was anonymised at this time. A combination of Microsoft Excel and NVivo 12 was used to store and analyse the interview data. The data were initially analysed using thematic analysis for inductive exploration and development of an initial coding framework. This approach was taken to ensure findings were driven from the data and were not manipulated to fit the theory. To assist in the interpretation of data and to ensure that contextual factors were adequately included in the analysis, CM independently recoded pseudonymised transcripts using an independently developed and validated NPT Coding Manual [14]. In the final stages, findings were mapped onto the NPT framework. There were no themes that did not map onto the four constructs of NPT and subsequent interpretation of findings ensued, including highlighting patterns, meaning, and associations.

Iterative discussion about coding and analysis took place with DF, MH and CM. Quotes were chosen to provide evidence of interpretation of the data and provide a deeper understanding of the interviewees’ thoughts and beliefs.

## Results

A total of 23 participants were interviewed (Table 2). A surgeon and two managers from two separate sites were unresponsive to email invitations, and a surgeon and a manager were not identified from two further sites.

**Table 2:** Number of patient and stakeholder interviews conducted per site

Site no.	Patient	BCN	Surgeon	Manager
1	0	1	1	1
2	2	2	0	0
3	1	2	1	1
4	0	2	0	0
5	3	1	1	0
6	2	2	0	0
Total	8	10	3	2

The following four themes reflecting influencers to implementation were identified and mapped onto NPT constructs (Table 3): Group CBT met a need for non-medicalised hot flushes/night sweats treatment, Surgeons, BCNs and patients were keen on the provision of nurse-delivered CBT to meet patient demand, BCNs were a trusted source for the delivery of CBT but need multi-level support to operationalise the work, Some BCNs and patients continued to benefit beyond the intervention.

**Table 3:** Themes mapped onto NPT constructs

NPT construct	Theme	Theme description
Coherence	Group CBT met a need for non-medicalised hot flushes/night sweats treatment	The intervention was perceived to have the ability to meet a patient need
Cognitive Participation	Surgeons, BCNs and patients were keen on the provision of nurse-delivered CBT to meet patient demand	There was mutual agreement amongst implementers that BCNs should lead the intervention
Collective Action	BCNs were a trusted source for the delivery of CBT but need multi-level support to operationalise the work	Multi-level factors enable the operational work (e.g. clinic cover)
Reflexive Monitoring	Some BCNs and patients continued to benefit beyond the intervention	There was a lasting application of the CBT skills learnt

### Coherence: Group CBT met a need for non-medicalised hot flushes/night sweats treatment

Despite working in a resource-constrained environment, the pressing need presented by the volume and severity of symptoms presented by patients motivated staff to implement a non-medical treatment to address the impact of hot flushes and night sweats.

“..patients would come in with these problems all the time, I can’t sleep, the hot flushes, and you feel so helpless and you want to give them information.” BCN

The staff has very little, if any, prior CBT exposure, but from the trial information and training understood it had the potential to be a solution to a currently unmet need.

Patients were also unfamiliar with CBT and initially felt sceptical due to a lack of understanding of how CBT works and their involvement in group sessions, despite this, they were willing to take part because they felt frustrated by previously ineffective, unsuitable or temporary treatment options and the impact that hot flushes/night sweats were continuing to have on their quality of life.

“I did think how on earth is CBT going to - I didn’t know very much about CBT..it’s a type of counselling, isn’t it? How on earth are you going to do that in a group? Are you going to be expected to talk about your childhood or something? I don’t know [chuckling].” Patient

“..It was anything at that point. By the time I was asked..when a nurse approached me, I’d had enough of not being able to sleep.” Patient

Despite the staff’s understanding and optimism towards the intervention, recruitment was not as successful as they had initially hoped. From the interviews conducted, the causes of suboptimal recruitment were believed to be because of the level of commitment required by patients, mainly the weekly meeting face-to-face at a specific time. It was also noted by many that the allocation of half the women to CBT and half to a control arm wouldn’t happen in a routine practice service and that out of a trial setting non-randomised recruitment to the CBT sessions would be quicker.

Some staff also suggested that group-based sessions, as opposed to one-to-one, could deter some people from taking part. Despite this, staff felt overall there was merit in providing CBT in a group setting because of its ability to foster a supportive and encouraging environment for the patients and be resource efficient for the hospital organisation.

“..we have so many people that phone up with hot flushes and night sweats and not being able to cope, it can take you an hour on the phone and if we’re getting three or four of these a week, that’s literally four hours out of your day where you could be doing a session that could..actually save time in the long-run.” BCN

Staff expectations were confirmed by patients who reported feeling anxious about the prospect of being in a group due to the embarrassing nature of hot flushes/night sweats, but later felt a great benefit to being able to give and receive support through shared experience.

“..it’s intimidating to go to a group session. I mean, a lot of people do find hot flushes so embarrassing, and they’re really self-conscious.It’s kind of like catch 22; you want to go because you want to get the help, but then you think, oh, God, everyone’s judging me. Then it’s like, like-minded people, everyone’s in the same boat.”. Patient

“I think being in a group is essential.” Patient

### Cognitive participation: surgeons, BCNs and patients were keen on the provision of nurse-delivered CBT to meet patient demand

Throughout the interviews, it became apparent that each staff group had a role in enabling the practices required to organise and do the work needed to implement group CBT.

Although only three surgeons were interviewed, all viewed CBT as a promising option to help cancer-free patients live a good quality of life and were supportive of the concept despite a lack of direct impact on their work.

“I think most clinicians wouldn’t really be fussed, because it doesn’t mean it’s something that affects them. However..I think it’s very important that all issues should be dealt with and, if they are as a result of treatment that you’ve instituted, it’s even more important, and it’s just a case of the best and most effective way of doing that.” Surgeon

“I think it’s really important. It’s all very well saying to a woman, ‘I’ve cured you of cancer’, but if you’ve given them a really rotten quality of life in doing so, then you’ve not really done your job very well. I always say to my patients, ‘It’s that balance about making sure that you’re cancer-free, but also making sure that you’ve still got a life at the end of it’.” Surgeon

Surgeons, as principal investigators, were responsible for the oversight of MENOS4 for recruiting sites, and in practice, their support is important because they reported being involved in local decisions relating to staff capacity and resource management. When prompted about how group-based CBT could be implemented into routine practice, surgeons shared the potential for patient-driven, as opposed to an organisation-driven, service. This way of working was supported by some patients who perceived a lack of need for health services at later routine follow-up appointments. However, patients and BCNs warned that a higher level of supervision might be needed for less communicative and proactive patients to ensure they were not suffering in silence. This concern was validated by patients worrying that they may come across as unappreciative if they expressed the trouble they were having with their hot flushes/night sweats, driven by ‘survivor’s guilt’ [15] and a juxtaposition to their gratitude for being cancer-free.

“I think a lot of people would tend to think that because you’re alive after having a diagnosis of cancer, then you know, you’re making a fuss about menopausal symptoms..you’re complaining about them when you’re actually alive and other people are not.” Patient

Moving on from clinical oversight and approval, across all sites, the BCNs took responsibility for the work needed to implement the RCT and deliver the CBT sessions. This included logistical organisation (e.g. booking and setting up rooms), negotiating time in existing job plans and mental preparation for the delivery of the CBT. When asked who should have responsibility for running the CBT service, all stakeholders believed BCNs were the most appropriate staff group. BCNs felt motivated to deliver the CBT sessions because they experienced personal and professional benefits from upskilling in a new area and an emotional reward felt for delivering care to patients. Managers had an awareness of MENOS4 and little operational involvement, and surgeons expressed a natural fit with existing nurse-led cancer survivorship service. Overall, the crucial role of identifying, engaging and delivering to patients was taken on by the BCNs.

“I’m aware of it. I’m the named PI for that here, but it’s the breast care nurses who’ve really been running the show, and I’ve let them get on with it and be happy. I’ve been really happy that they’ve been doing it, actually.” Surgeon

When the same question was posed to participants, indifferent to whether the BCNs were already known to them, they believed BCNs were best placed to deliver group CBT because of their knowledge of breast cancer and experience with patient journeys.

“I think it was really good to be done by the nurses, because they really understood exactly what we’d all gone through. They’d seen it….So, they really knew what we were going through, what were the problems, and how people dealt with it, so they were very familiar, and I thought that was important.” Patient

### Collective action: BCNs were a trusted source for the delivery of CBT but need multi-level support to operationalise the work

Data collected from the interviews explored the resources and practical nuances of delivering the intervention. CBT delivery was accepted by BCNs because it fitted within the scope of current work and resonated with the reason they became a nurse. The CBT training and supportive materials (e.g. relaxation audio recording) received by BCNs fulfilled their needs to confidently deliver the service, but for the intervention to be adopted into routine care, support is necessary at multiple levels to overcome implementation barriers.

The following responsibilities were identified for the various peer groups to enable the BCN to implement and deliver the service (Table 4).

**Table 4:** Peer group implementation responsibilities

Peer group	Role
Manager	Support the BCNs in understanding what their role and associated responsibilities are and work collaboratively to develop an effective business case to board members.
Medic	A key decision maker for implementing a new service and facilitates discussions that enable BCNs to streamline their duties so that work can be re-directed work to administrative/ other supporting members of staff.
Breast Care Nurse	Reflect on current responsibilities to facilitate the re-direction of work and ensure additional work is not taken on that is not congruent with their role. Communicate the need for new services to managers and operationalise the work.
Patient	Create awareness of/demand for a service and communicate needs (or lack of) to optimise patient care.

Regarding the actual work done to deliver the intervention, a mixed approach to advertising MENSO4 was seen to be most effective by BCNs. BCNs, with the support of some surgeons, actively identified patients during appointments, and in parallel used passive paper-based advertising (e.g. leaflets and newspaper advertising) to increase patient reach, which was believed to be effective because they experienced subsequent enquiry from patients. When patients were asked what patient approaches they felt might be effective, some recommended using participant quotes and word of mouth through the cancer community and utilising cancer-support networks to educate and increase awareness of group CBT for those with a potential lack of understanding and familiarity with CBT, i.e. a psychological therapy for physical symptoms.

“Somebody who’s been through a similar situation to them, and then has been on this training and has really seen the benefits from it. I think that’s always a good endorsement..” Patient

“My friend, who’s a little bit older than I am.. had seen it on the news. And her suggestion was, ‘you go on the trial. You go and see about it and you can tell me what I need to do’.” Patient

Although BCNs held primary responsibility for the work done to put the intervention into practice after the identification of a potential participant research staff conducted trial procedures including informed consent and baseline data collection. Although this helped with BCN staff capacity, some felt this loss of continuity created a disjoint in communication with patients that could have been a recruitment barrier. It was suggested that in routine practice they would delegate similar tasks to closer department colleagues so they would be able to maintain involvement and avoid such issues.

Beyond trial-imposed patient and inter-department communication, several organisational barriers to adoption were identified, the most common being resource and capacity.

Resource is tightly constrained in the NHS, and although BCNs are deemed to have autonomy, MENOS4 at times created friction with current work demands put upon them by clinics and MDT meetings. Conflicting demands were temporarily managed by reliance on peer support or authoritative buy-in (senior manager or medic) to release them from clinic and support capacity for individual agency. Department managers were supportive of BCN-led services with the caveat that they needed to continue managing their existing workload. As a compromise, BCNs often completed work for MENOS4 in their own time because they deemed the intervention an important part of patient care.

“There’d be no way to take work time, just because of the pressures we’ve got with clinics and just general, our workload is just… Everybody’s is immense, isn’t it, so you couldn’t take time out of your day to prep, no.” BCN

To adopt group CBT as a routine service, medics, managers and BCNs would work together to create a business case that would need to show that the investment in the resource is worthy of the clinical outcome.

BCNs were concerned that NHS decision-makers might deem CBT a “luxury” or “flowery stuff”. BCNs believe services at the hospital should be patient-led and backed by research and felt that hospital organisations are led by a metric-driven prioritisation process that demotes the precedence of “alternative” and psychological therapies.

Managers and surgeons emphasised similar concerns for adoption, including how to communicate a favourable financial outcome that would be appealing to organisational board members.

“I haven’t got any problem writing something for the Board to consider..like I said the financial impact would be the backfilling of the clinical teams, their time and whether or not the organisation is prepared to support that.. From a quality perspective, I think that they would but from a financial element of it, depending on the cost they may not“ Manager

Many sites offered cancer-survivorship services and complementary therapies, but none focused on the specific management of hot flushes/night sweats. To help manage resources and the sustainability of the service (helpful for the business case), nearly all BCNs suggested condensing and integrating group CBT into existing services.

“We’ve been talking about, the Move Forward programme is an afternoon for four weeks. We have talked about maybe, if this is taken up by the NHS, we could slot it on to the end for those few that - because then you’ve got a captive audience.” BCN

An alternative suggestion for secondary care implementation involved integrating services with primary care and community services to identify patients and distribute the impact on resources, respectively, and was thought to have the potential to hold mutual benefit for service providers and those in need of hot flushes/night sweats treatment.

“I mean it could be something that we could approach the CCG for... That’s an option and that’s certainly something that we would look at if we were doing an appraisal of it to take it forward. We would look at alternative of joint working or if there’s somebody in the community that we could actually tap into…. if we approach the CCG and said, ‘Okay then how about us going 50/50? We’ll share it with you, we’ll share the cost” Manager

When trying to plan the group CBT sessions, scheduling a group time was one of the greatest challenges because of existing BCN commitments and the heterogeneity of the population (e.g. age, employment status, dependants). Groups held during the day did not capture working patients, but groups held before or after 9am and 5pm respectively, had traffic and family commitments to contend with.

“We were doing it at the end of a day. 1) Because we thought we’d get more of the ladies to come, but 2) during the day, you’ve got clinics and other things going on.” BCN

“I think, if they’re younger and they’re working, I think the time commitment’s difficult for them; and I think for - certainly for one of ours in the group - it was very difficult timing; because it was half past four ‘til six - which you would think was quite a good time - but [city name], if you’ve got to get anywhere at half past four, it’s absolute gridlock.” Patient

In addition to having the potential to appease organisational board members, integrating with existing cancer-survivorship services was seen as a potential solution to overcome practical scheduling issues.

### Reflexive monitoring: some BCNs and patients continued to benefit beyond the intervention

Overall, group CBT was seen to provide benefits to both the staff and patients. For BCNs, CBT skills learnt were being transferred into different contexts for patient benefit.

“To be perfectly honest, I have to say both myself and [nurse colleague name] have found that with some of our really anxious patients, and also with our patients with menopausal symptoms, we’re using the stuff.” BCN

“Yes, I use that a lot for lots of things. When I’m feeling stressed as well, I use that breathing, and the ‘it’s a thought, not a fact’, lots of things like that. There’s been lots of stress in my life in the last couple of weeks, so that’s been really, really helpful as well.” Patient

Outside of the constraints of a trial, BCNs would like to deliver CBT in a less clinical and more informal environment. Although patients felt the hospital was a suitable place, BCNs suggested creating space different to other parts of the hospital (e.g. soft furnishings) could encourage engagement and create a more positive association with the service.

“I do think not necessarily happening in a hospital environment is the best... Also, patients coming back to this environment, it’s like revisiting what’s happened and they don’t like coming on annual visits never mind coming for a couple of weeks. I think if we could change the…One of the ladies did say about the layout the one day, didn’t she? She said, ‘Oh, it’s like being back in school because the one room we had.” BCN

Finally, patients perceived MENOS4 to be of great benefit because it added structure, social support, perspective, materials for reflection and a means to identify triggers and manage hot flushes/night sweats, which ultimately led to a sense of control and increased quality of life.

“Life, completely, yes! It’s given me control back. I mean, that sounds really silly, but it has. It’s kind of - my new normal isn’t different to my old normal, but no, I’ve got control back, and that’s what CBT did.” Patient

## Discussion

This qualitative process evaluation revealed that nurse-delivered group CBT is acceptable to stakeholders and has the potential to be implemented as an NHS-provided service if BCNs are given multi-level support. This support appears to depend on the extent to which services are receptive to and will adopt, an intervention that may be considered a luxury that might not have a high impact on their performance metrics.

On an individual level, BCN-led CBT facilitated closer engagement with patients and developed their professional role. The importance of this is facilitated by existing literature that demonstrates that the concept of partnership was integral to patients’ self-control and ownership of symptom management and the nurses’ ability to meet the needs of patients, elicit clinical information and coordinate care [16, 17].

Some patients initially began the group-based CBT with scepticism due to a lack of understanding and familiarity, but with experience, they highlighted that it was an important part of the service that fostered peer support and encouragement, showing that this method of delivery is acceptable.

Surgeons, although they acknowledged the importance of the implications of hot flushes/night sweats, are not actively treating women who would be considered cancer-free. BCNs held primary responsibility for the initial implementation and operationalisation of work at the cost of either giving additional workload to peers or completing tasks outside of work hours. Staff capacity was highlighted as a key barrier throughout the interviews, mirroring issues in implementing CBT services by psychologists, with managers negotiating clinical trade-offs within the constraints of set financial budgets [18].

The verbal support from senior staff creates a conflicting message that initially suggests BCNs have the individual agency to trial and lead new patient services, but with little to no organisational investment and friction with an existing workload, this becomes pseudo-agency. Agency is necessary for BCNs to have the resources and ability to act on their own choice. A lack of agency limits autonomy, which is regarded as an essential element of professional status [19].

Consequently, there may be friction between BCNs’ individual values (wanting to provide patient-centred services) and the expectations of their managers and employers (e.g., clinics and team meetings). Cohesion with the organisation, resource, and multi-level support are commonly reported factors in the implementation of nurse-led interventions in different countries and settings (e.g., care homes) for different patient groups [20, 21], emphasising the importance of a culture of support when implementing nurse-led interventions.

A major concern by staff stakeholders was the priority-setting values of secondary care organisations which meant group CBT was unlikely to be supported if it did not at least meet financial equipoise, which is not the same priority-setting values BCNs share. These areas of conflict are especially important findings, given that BCNs working in their own time is not sustainable and not being able to work to their values is a major cause of burnout in Oncology nurses [22].

To mitigate this area of tension, alternative methods may be a viable option, such as integration with existing services or the use of alternative staff. It should be noted however that a major motivator for patients to receive a treatment they understood little about was the belief in the BCNs as a trusted source. Decision-makers implementing the service should be cautious when considering alterations to advertisement and delivery to mitigate the impact on patient uptake and engagement. In contrast to existing literature, none of the interviewed stakeholders showed concern for nurse knowledge and competencies, which could suggest that the CBT training and material received were sufficient for BCNs to feel confident in delivering the service and apply newly learnt skills both personally and to other patients for menopause and anxiety, which implies there is a wide range of application for CBT.

Finally, pro-active patient demand for long-term follow-up, as opposed to scheduled appointments, was suggested by some to relinquish some capacity; however, some patients in this group express ‘survivors guilt’ and may not proactively communicate side effects of treatment for fear of seeming unappreciative and ungrateful for being cancer-free. To combat these concerns, links with primary care, trusted advertisement (e.g. cancer networks) and targeted follow-up for less expressive patients should be considered.

In summary, based on this NPT-informed process evaluation the findings suggest group CBT be a needed and accepted means to treat hot flushes/night sweats in breast cancer survivors. Strategies facilitating the adoption of the service into hospital Trusts included incorporating group CBT within existing survivorship services and re-directing departmental workload to provide BCNs with the resource to deliver the service. However, to achieve adoption and sustainability several coordinated patient, staff and organisational level factors need to be negotiated, highlighting the challenges of implementation within a complex resource-constrained healthcare system.

### Limitations

The study had limitations in that the numbers of surgeons and managers were very small due to a lack of availability. Secondly, we examined the intentions and perceived feasibility of implementing CBT into normal practice within a clinical trial which may have affected managers’/surgeons’ views about implementation. Similarly, at the time the participants did not know the outcomes of the trial, which were very positive, at the time of interview.

## Conclusion

All stakeholders felt that group CBT was appropriately delivered by the BCNs and was wholly beneficial at an organisational, staff and patient level. The primary barrier to routine care adoption is adding the service to already stretched BCN time; however, with multi-level support, CBT to manage hot flushes/night sweats in breast cancer survivors is feasible despite current constraints.

### Electronic supplementary material

Below is the link to the electronic supplementary material.

Supplementary Material 1

Supplementary Material 2

Supplementary Material 3

Supplementary Material 4

## Data Availability

The datasets generated and/or analysed during the current study are not publicly available to protect the identity of the participants but are available from the corresponding author on reasonable request. The topic guides used to inform the interviews are available as supplementary material. Please direct all data enquiries to the corresponding author.

## Abbreviations

BCN: Breast Care Nurse
CBT: Cognitive Behavioural Therapy
NHS: National Health Service
NPT: Normalisation Process Theory
