# Supplementary material for: Implementing a nurse-delivered cognitive behavioural therapy intervention to reduce the impact of hot flushes/night sweats in women with breast cancer: a qualitative process evaluation of the MENOS4 trial
Source: BMC Nurs. 2023 Sep 15;22:317. doi: 10.1186/s12912-023-01441-3 (PMC10503156; doi:10.1186/s12912-023-01441-3)
Supplement: Supplementary file 2 — Supplementary Material 2 [file 12912_2023_1441_MOESM2_ESM.pdf]

MENOS4 Medic and Managers Topic Guide

- Re-introduce self and purpose of interview
    - Ask participant if they have had a chance to read the information sheet and ask if they have any questions about the study.
- Remind the participant
    - Their responses will be kept confidential; any direct quotes will not be used to identify them as an individual.
    - They can change their mind about taking part in the study, can stop the interview at any time or decline to answer a question.
    - Remind them that the interview will take approximately 45 minutes.
    - Confirm consent and permission to record.

- Please describe your role
- What surgeries, clinics, wards do you work across?
- What relationship do you have with BCNs?
- What role/s do BCNs have?
- Does your remit include treatment of HFNS?
- What enables your service to address HFNS in your work?
- What prevents your service from addressing HFNS in your work?
- Are there any current approaches used in your services for helping patients with HFNS?
- Have you spoken to the BCNs who have taken part in this trial about the group CBT?
- What do you think is the value of CBT for HFNS in your own words?
- If we find that group CBT delivered by BCNs is effective, how would you present the case for running this intervention in routine practice? What would that case look like?
- Do you envisage the need for extra human (time, staffing) and physical (room booking, consumables) resources to run the group CBT?
- Would you be able to release the BCNs from other duties? How would you plan to address this? Is there any contingency plan in place?
- Is there anything else you'd like to share?
