# Supplementary material for: Implementing a nurse-delivered cognitive behavioural therapy intervention to reduce the impact of hot flushes/night sweats in women with breast cancer: a qualitative process evaluation of the MENOS4 trial
Source: BMC Nurs. 2023 Sep 15;22:317. doi: 10.1186/s12912-023-01441-3 (PMC10503156; doi:10.1186/s12912-023-01441-3)
Supplement: Supplementary file 3 — Supplementary Material 3 [file 12912_2023_1441_MOESM3_ESM.pdf]

MENOS4 Nurse Topic Guide

- Re-introduce self and purpose of interview
    - Ask participant if they have had a chance to read the information sheet and ask if they have any questions about the study.
- Remind the participant
    - Their responses will be kept confidential; any direct quotes will not be used to identify them as an individual.
    - They can change their mind about taking part in the study, can stop the interview at any time or decline to answer a question.
    - Remind them that the interview will take approximately 45 minutes.
    - Confirm consent and permission to record.

- What is your role as a BCN?
- How did you find the training you received? How did you find learning about CBT in this way? Did you develop and learn any new skills in order to complete the training?
- What is your understanding of treatments for HFNS now?
- Do you have a new understanding of how HFNS are experienced by breast cancer patients?
- How do you see CBT for HFNS fitting in to your wider work?
- What was your experience of delivering this intervention to patients? Was it clear to you what you were required to do and how? Did you develop and learn any new skills in order to deliver the training?
- In light of your experience of delivering the training to patients, what would enable you to offer group CBT to your patients?
- What would prevent you from offering group CBT for women with HFNS in your work?
- Please describe the value of CBT for HFNS in your own words?
- Did you have a plan in place for setting up the CBT sessions? How did this plan work?
- What were the team working processes for implementing the CBT with respect to your managers?
- What were the team working processes for implementing the CBT with respect to your other colleagues?
- How would you be able to incorporate the work needed to deliver the CBT into your workload?
- Were there any features of the intervention that made delivering the session particularly acceptable to you?
- Were there any aspects of the delivery that were unsuitable for you?
- Is there anything else you'd like to share?
