# Supplementary material for: Implementing a nurse-delivered cognitive behavioural therapy intervention to reduce the impact of hot flushes/night sweats in women with breast cancer: a qualitative process evaluation of the MENOS4 trial
Source: BMC Nurs. 2023 Sep 15;22:317. doi: 10.1186/s12912-023-01441-3 (PMC10503156; doi:10.1186/s12912-023-01441-3)
Supplement: Supplementary file 4 — Supplementary Material 4 [file 12912_2023_1441_MOESM4_ESM.pdf]

MENOS4 Patient Topic Guide

- Re-introduce self and purpose of interview
  - Ask participant if they have had a chance to read the information sheet and ask if they have any questions about the study.
- Remind the participant
  - Their responses will be kept confidential; any direct quotes will not be used to identify them as an individual.
  - They can change their mind about taking part in the study, can stop the interview at any time or decline to answer a question.
  - Remind them that the interview will take approximately 45 minutes.
  - Confirm consent and permission to record.

- What is your history of HFNS? How are your HFNS now?
- How would you describe the value of CBT for your HFNS, in your own words?
- What was it about the CBT groups that made you want to attend?
- How did you find out about the CBT treatment? Why did it appeal to you?
- How suitable is it that BCNs run CBT groups for HFNS to patients?
- Were there any aspects of the delivery by BCN that were unsuitable for you?
- Did you have the opportunity to think and talk about your experience of HFNS? Did the CBT groups affect your perception of HFNS?
- Did attending the groups encourage you to think differently to your approach to managing HFNS episodes?
- What would you say were the main benefits that you have had from having the group CBT?
- What changes (if any) have you made to your 1) behaviour and 2) lifestyle as a result of your participation?
- How did you manage finding sufficient time to attend the sessions?
- Were there changes that you had to make to fit it into your daily routine? If so, in what ways? If no, could you explain why?
- Is there anything else you'd like to share?
